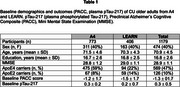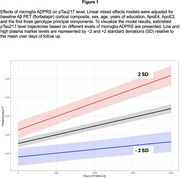# Microglia‐specific Alzheimer's disease polygenic risk score predicts longitudinal increase in plasma tau and faster cognitive decline in cognitively unimpaired older adults

**DOI:** 10.1002/alz70856_106441

**Published:** 2026-01-10

**Authors:** Brahyan J Galindo Mendez, Ling Teng, Gad A. Marshall, Lei Liu, Jasmeer P. Chhatwal, Timothy J. Hohman, Richard Mayeux, Philip L. De Jager, Robert A. Rissman, Keith A. Johnson, Reisa A. Sperling, Hyun‐Sik Yang

**Affiliations:** ^1^ Center for Alzheimer Research and Treatment, Department of Neurology, Brigham and Women's Hospital, Boston, MA, USA; ^2^ Mass General Brigham, Boston, MA, USA; ^3^ Harvard Medical School, Boston, MA, USA; ^4^ The Broad Institute of MIT and Harvard, Cambridge, MA, USA; ^5^ Department of Neurology, Vanderbilt Memory & Alzheimer's Center, Vanderbilt University Medical Center, Nashville, TN, USA; ^6^ Department of Neurology and the Taub Institute for the Study of Alzheimer's Disease and the Aging Brain, Columbia University Irving Medical Center, New York, NY, USA; ^7^ University of Southern California, San Diego, CA, USA

## Abstract

**Background:**

Alzheimer's disease (AD) is a highly heritable neurodegenerative disorder, and human genetics have strongly implicated microglia (Mic) in AD pathogenesis. Leveraging our novel method to derive cell‐type‐specific AD polygenic risk scores (cts‐ADPRS), we examined their association with longitudinal plasma‐phospho‐tau‐217 (pTau217) and cognition in cognitively unimpaired (CU) older adults.

**Methods:**

We analyzed longitudinal data from a secondary AD prevention trial (A4; CU with elevated+Aβ and LEARN; CU with sub‐threshold ‐Aβ). cts‐ADPRS were derived and standardized using prior published method by (1) excluding APOE and selecting top 10% of genes specifically expressed in each major brain cell type (excitatory neurons, inhibitory neurons, astrocyte, microglia [Mic], oligodendrocyte, oligodendrocyte progenitor cells) and (2) deriving each cts‐ADPRS using the variants within these genes ± 30 kb. We analyzed the relationship of each cts‐ADPRS with longitudinal change in pTau217 and Preclinical Alzheimer Cognitive Composite (PACC). We used linear mixed effect models (LMEM) adjusted for baseline Aβ PET (florbetapir) cortical composite, age, sex, *APOE* ε4/ε2, years of education, first three genotype principal components, and their time interaction terms. We extracted adjusted random slopes of pTau217 and PACC from LMEM and, performed a mediation analysis to examine the relationship among cts‐ADPRS, pTau217, and PACC.

**Results:**

We included 1179 CU subjects (*n* = 474 females (40%), 70.9 ± 4.5 years old) of European descent who had pTau217 and PACC. Mic‐ADPRS was associated with a longitudinal increase in pTau217 (beta = 13.9 x, SE = 2.4 x, *p* =  <0.001) while none of the other cts‐ADPRS were (all *p* >0.05). Mic‐ADPRS was also associated with faster PACC decline (beta= ‐15.0 x, SE = 1.8 x, *p* =  < 0.001). Mediation analysis suggests 42% of Mic‐ADPRS–PACC association is mediated by increased pTau217.

**Conclusion:**

Our findings suggest AD heritability localizing to microglial genes and contribute to increased soluble pTau217 release at a given Aβ burden, which in turn mediates the association between microglial AD genetic risk and cognitive decline. Our results are consistent with previous studies implying microglia in Aβ‐related tau accumulation and indicate that much of microglial impact on cognitive decline may occur through accelerated tau pathology in CU older adults.